# Best Reply Player Against Mixed Evolutionarily Stable Strategy User

**DOI:** 10.1007/s11538-021-00980-7

**Published:** 2021-12-24

**Authors:** József Garay, Tamás F. Móri

**Affiliations:** 1 Institute of Evolution, Centre for Ecological Research, Konkoly-Thege Miklós út 29–33, Budapest, 1121 Hungary; 2 ELKH–ELTE Theoretical Biology and Evolutionary Ecology Research Group, Loránd Eötvös University, Pázmány Péter sétány 1/c, Budapest, 1117 Hungary; 3 Alfréd Rényi Institute of Mathematics, Reáltanoda u. 13–15, Budapest, 1053 Hungary

**Keywords:** Dynamical player, Iterated game, Mixed strategy, Markov model, Population game, 91A05, 91A22, 92D15

## Abstract

We consider matrix games with two phenotypes (players): one following a mixed evolutionarily stable strategy and another one that always plays a best reply against the action played by its opponent in the previous round (best reply player, BR). We focus on iterated games and well-mixed games with repetition (that is, the mean number of repetitions is positive, but not infinite). In both interaction schemes, there are conditions on the payoff matrix guaranteeing that the best reply player can replace the mixed ESS player. This is possible because best reply players in pairs, individually following their own selfish strategies, develop cycles where the bigger payoff can compensate their disadvantage compared with the ESS players. Well-mixed interaction is one of the basic assumptions of classical evolutionary matrix game theory. However, if the players repeat the game with certain probability, then they can react to their opponents’ behavior. Our main result is that the classical mixed ESS loses its general stability in the well-mixed population games with repetition in the sense that it can happen to be overrun by the BR player.

## Introduction

In game theory, the Nash equilibrium is an optimal situation where neither player can benefit by changing strategy while her opponent keeps hers unchanged. A more general concept of game theoretical solution is the correlated equilibrium (Aumann 1974, 1987). It is based on the assumption that players choose their actions according to their observation of the value of the same public signal, e.g., they may keep monitoring their opponent’s past performance. This raises the question whether reactive players can overcome Nash players, from the view point of evolutionary game theory.

Consider a sufficiently large asexual population with nonoverlapping generations, where the interaction is well mixed (i.e., in each round, the probability of interactions is proportional to the relative frequency of the phenotypes). For this selection situation, based on the Darwinian tenet, Maynard Smith and Price (1973) introduced the intuitive definition of monomorphic evolutionary stability: a phenotype is evolutionarily stable if a rare enough mutant cannot invade the resident monomorphic population displaying this phenotype. For pairwise interactions, the general formalization of this verbal definition reads as follows. Let *W*(*X*, *Y*) denote the average benefit of phenotype *X* when interacting with phenotype *Y*, Then resident phenotype *X* is said evolutionarily stable, if for arbitrary mutant phenotype *Y* with sufficiently small relative frequency $\varepsilon \in (0,1)$ we have

1 $$\begin{aligned} (1-\varepsilon )W(X,X)+\varepsilon W(X,Y)>(1-\varepsilon )W(Y,X)+\varepsilon W(Y,Y),\quad 0<\varepsilon <\varepsilon _0. \end{aligned}$$

Note that the smallness bound $\varepsilon _0$ may vary with *Y*.

Starting from a symmetric matrix game, let $S=\{s_{1},\dots ,s_{d}\}$ denote the set of pure strategies, then the phenotypes are described by mixed strategies, i.e., by probability distributions over *S*. In this case, the phenotype using the mixed strategy *p* will also be denoted by *p* if it does not lead to misunderstanding. Suppose that the average benefit of the interaction between individuals is given by a matrix game, i.e., $W(p,q)=pAq$, where $A\in {{\mathbb {R}}}^{d\times d}$ and *p*, *q* run over the $(d-1)$-dimensional simplex ${\Sigma }_{d}= \big \{x\in {{\mathbb {R}}}^d: \forall x_i\ge 0,\ \sum _{i=1}^d x_i= 1\big \}$. In this model, the above general definition of ESS (1) reads as follows: $p^{*}$ is an evolutionarily stable strategy (ESS) if a small enough size of mutant population implies that for every possible mutant strategy *q* we have

2 $$\begin{aligned} (1-\varepsilon )p^{*}Ap^{*}+\varepsilon p^{*}Aq>(1-\varepsilon ) qAp^{*}+\varepsilon qAq,\quad 0<\varepsilon <\varepsilon _0, \end{aligned}$$

which yields the well-known definition of ESS for matrix games (see Maynard Smith and Price 1973; Hofbauer and Sigmund 1998).

We emphasize that here we only concentrate on symmetric games, where each player has the same strategy set and the same payoff matrix, since, as it is well known, there is no mixed ESS in asymmetric games (Selten 1980, 1983).

### Remark 1

Although Maynard Smith and Price (1973) considered well-mixed interaction in (2), their idea can be used for iterated games too. (By iterated game, we mean that the same opponents play the same matrix game in a huge number of times.) Indeed, if each individual is paired randomly with another individual from the population (i.e., the pair formation is well mixed), each pair plays a large but the same number of games with each other (i.e., the payoffs are defined as the limit of the average payoffs per round), and each player can only use a genetically fixed mixed or pure strategy in all rounds, then the definition of ESS (2) remains valid. We emphasize that there is an essential difference between the well-mixed (fixed) pair formation and the well-mixed interaction, since in the latter case each player gets a new random opponent from the whole population from round to round, so there is no possibility to either synchronize their actions or react to the partner’s strategy.

However, it is well known that the properties of iterated games are very different from those of one-shot games (van Damme 1987). On the one hand, Cressman (1992) discussed the twice repeated one-shot game where there are two strategies. By analyzing the ESSs of this eight-strategy two-round game, he found that using the one-shot ESS in each round was seldom an ESS in the two-round game. In fact, if the one-shot ESS is a mixed strategy, it is never an ESS of the two-round game (personal communication). Based on that, here we consider repetitive games of randomly paired individuals, and only concentrate on the one-shot mixed ESS in the hawk-dove game.

On the other hand, pair formation can also be a behavior. Pacheto et al. (2006a; b) studied the consequences of dynamical linking, where the number of repetitions of the interactions between two individuals depends on the payoff from the given interaction, and they found the natural selection to favor cooperation over defection.

Furthermore, there are other types of mutation than the strategy mutant. In the resident population of the standard behavior (“maximization of own payoff”), the mutants may have different other regarding preferences (e.g., “to be better than the average,” see Garay and Varga 2005), morality (e.g., “to do the right thing,” see Alger and Weibull 2012, 2013, 2019; Weibull 1997), or amorality (e.g., envy “punish the successful,” see Garay and Móri 2011). In these papers, the evolution operates on the different “player types” and not on different strategists only. Now, we also consider two different types of player: the resident is able to use mixed strategy, while the mutant can use the best reply strategy. We care which one will win on evolutionary time scale (i.e., we are looking at the “end of evolution” across generations), and this is the reason why we consider the uninvadability inequality (1) the foundation of the present paper.

Iterated games are widely used in economics and evolutionary game theory. *Firstly*, remember that the notion of mixed strategy is built on iterated games between two players. *Secondly*, there exist important studies on evolutionary stability in iterated games. In particular, from the literature of the iterated prisoner’s dilemma game (e.g., Axelrod and Hamilton 1981; Hilbe et al. 2013), it is well known that the iteration of the one-shot ESS in the prisoner’s dilemma is not an ESS in the iterated prisoner’s dilemma (e.g., Bendor and Swistak 1995; Boyd and Lorberbaum 1987; Farrell and Ware 1989; García and van Veelen 2016). Another example is the iterated survival game, where the altruism is evolutionarily stable (Garay and Varga 2011; Wakeley and Nowak 2019). *Thirdly*, the iterated hawk–dove game has already been studied (e.g., Houston and McNamara 1991; Wolf et al. 2011; Morrell and Kokko 2003, 2005; Van Doorn et al. 2003). But, according to our knowledge, our selection situation, where best reply players compete with classical mixed ESS players, has not been investigated so far.

A mixed strategy is not reactive in the sense that a mixed strategy user randomly uses a pure strategy from game to game, independently of what her partner has done. Unlike a mixed strategy user, living things respond to stimuli, and if in the repetitive games a “reactive player” can consider her partner’s earlier used pure strategy as a stimulus, then the reactions of a reactive player can form a deterministic time series. In other words, reactive player does not necessarily use a mixed strategy. In general sense, firstly, in the framework of iterated prisoner’s dilemma game, e.g., the well-known “tit for tat” strategy (for other strategies see, e.g., Kendall et al. 2007) and the social norms (e.g., Ohtsuki and Iwasa 2006) belong to the set of reactive strategies. Secondly, the reactive player can imitate or learn (e.g., Hofbauer and Sigmund 1998; Szabó and Hódsági 2016), when she can compare her payoff with other players’ payoff. In the second case, the reactive player’s behavior is given by a game dynamics.^1^ Now, we focus on the dynamical player as a special case of the reactive player, and our general question arises: Can a mutant dynamic player invade a population of mixed ESS users?

In population games when each player plays many rounds, there are two extreme interaction schemes, namely well-mixed interactions and iterated ones. The former is the basic assumption of the classical ESS. If the population size is large enough (say infinite), the probability that a fixed pair repeats the game is negligible. On the contrary, in an iterated game the two players keep on playing the game exclusively with each other for a very long (virtually infinite) time. However, there is another possibility halfway between these two extreme schemes, where the interaction between two players is repetitive (i.e., the mean number of repetitions between two players is positive but finite), but when the two players finish the interaction, they form new pairs with other players at random, in a well-mixed way. We will call this *a well-mixed game with repetition*.

One of the possible biological examples for a well-mixed game with repetition is the territorial behavior (Morrell and Kokko 2005), when the neighbors are fixed while the floaters randomly arrive to fight for the owners’ territory (Varga et al. 2020). Clearly, the game repetition opens lots of questions if the players can collect information about their partners and they have memory. For instance, if there is physical difference between players, after fight the loser can accept hierarchy (Van Doorn et al. 2003), which creates asymmetry in the game, based on different fighting abilities of players. Since we would like to avoid asymmetric conflicts, we consider the simplest kind of memory: in the next repeated game each BR player can only remember the pure strategy used by her opponent in the preceding game, but cannot recall the payoff or the outcomes of previous games^2^ Furthermore, all players have the same fighting ability (Morrell and Kokko 2003).

In what follows, we first concentrate on the iterated game, and then we are going to deal with well-mixed population games with repetition.

## Model and Results

### Iterated Games

Here, we consider best reply (see, e.g., Hofbauer and Sigmund 1998) as a reaction rule: this is the strategy yielding the most favorable outcome against the opponent’s move in the preceding game. The present question is whether a best reply player can invade a monomorphic classical mixed ESS population. Let us consider a symmetric $2\times 2$ game (anti-coordination, hawk–dove) with payoff matrix

$$\begin{aligned} A=\left( \begin{array}{cc} a_{11} &{} a_{12} \\ a_{21} &{} a_{22} \end{array} \right) =\left( \begin{array}{cc} c &{} b \\ c+a &{} 0 \end{array} \right) \end{aligned}$$

($a,b>0$) with the ESS $p^{*}=(\frac{b}{a+b},\frac{a}{a+b})$. The average payoff against $p^{*}$ is

$$\begin{aligned} W(p^{*},p^{*})=p^{*}Ap^{*}=(Ap^{*})_{1}=(Ap^{*})_{2}= \frac{(c+a)b}{a+b}. \end{aligned}$$

Now assume the game is played repeatedly, and consider the strategy for the repeated game that starts with a uniform random action and then plays in every round a best reply against the action of the opponent in the previous round. Call this strategy $\beta$. Then the (expected) payoff (per round) for $\beta$ against $p^{*}$ (playing in every round action *i* with probability $p_{i}^{*}$, independently of the past) is again

$$\begin{aligned} W(\beta ,p^{*})=\frac{(c+a)b}{a+b}. \end{aligned}$$

Remind that, against the mixed ESS, all mutants are neutral in the sense that mutants receive the same payoff as the ESS strategist does. Since $\beta$ against $p^{*}$ uses (in the long run) action 1 with probability $p_{2}^{*}=\frac{a}{a+b}$ and action 2 with probability $p_{1}^{*}=\frac{b}{a+b}$, we get

$$\begin{aligned} W(p^{*},\beta )=cp_{2}^{*}+a{p_{2}^{*}}^{2}+b{p_{1}^{*}}^{2}= \frac{ca}{a+b}+\frac{a^{3}+b^{3}}{(a+b)^{2}}. \end{aligned}$$

Finally, $\beta$ against $\beta$ either alternate between 11 and 22, or play 12 forever, or 21 forever. Thus

3 $$\begin{aligned} W(\beta ,\beta )=\frac{2c+a+b}{4}. \end{aligned}$$

For $c=0$, we have

$$\begin{aligned} W(p^{*},\beta )=\frac{a^{3}+b^{3}}{(a+b)^{2}}\ge W(\beta ,\beta )= \frac{a+b}{4} \end{aligned}$$

with strict inequality if $a\ne b$. In this case $\beta$ cannot invade $p^{*}$.

Now let $a<b$ and $c>0$ large. Then

$$\begin{aligned} W(p^{*},\beta )<W(\beta ,\beta ). \end{aligned}$$

Indeed, by elementary calculation we obtain that

$$\begin{aligned} W(\beta ,\beta )-W(p^{*},\beta )=\left[ c-\tfrac{3}{2}(b-a)\right] \frac{b-a}{2(a+b)}, \end{aligned}$$

thus, if $a<b$ and $c>0$, then $W(p^{*},\beta )<W(\beta ,\beta )$ holds if and only if $c>\frac{3}{2}(b-a)$. In this case

$$\begin{aligned} W(p^{*},p^{*})-W(\beta ,\beta )=\left[ c-\tfrac{1}{2}(b-a)\right] \frac{b-a}{2(a+b)}>0. \end{aligned}$$

For a numerical example, let $a=1,$ $b=3$ and $c=6$. We note that if *c* is allowed to be negative, then $b<a$ and $c<\frac{3}{2} (b-a)$ will also enable $\beta$ to invade $p^{*}$.

Hence, the memoryless ESS $p^{*}$ loses against the memory one strategy $\beta$. According to (1), the mutant best reply phenotype can replace the classical mixed ESS phenotype $p^{*}$, since, independently of the relative frequency $\varepsilon$ of the mutant, the average fitness of $p^{*}$ is lower than that of the best reply phenotype $\beta$ (cf. Cressman et al. 2020).

#### Remark 2

What will change if a small probability of error is allowed? By error, we mean that the player fails to use the strategy she is expected to: her strategy specifies to use pure strategy $s_i$ but she uses another $s_j$ $(j\ne i)$ instead. It may have several reasons; for example, the player intends to use a certain strategy, but, by error, uses another one, or the BR player may erroneously perceive the strategy used by her opponent in the preceding round. The source of error is irrelevant: the only requirements are that (i) the random event of error has to be of sufficiently small probability, and (ii) it has to be independent of the past and present of the process. Particularly it cannot depend on the strategy she otherwise would use.

Let $\beta ^{\prime }$ denote the best reply strategy with an error of probability $\delta$, i.e., in every game, independently, error occurs with the prescribed probability. In our $2\times 2$ example erroneous best reply means changing to ESS when playing with an ESS player, and to another cycle when the opponent is another BR player. Indeed, when her opponent is an ESS player, the BR player virtually uses the mixed strategy $q^{*}$ being just the opposite of the ESS in the sense that the probabilities (or equivalently, the pure strategies) are interchanged: $q_1^{*}=p_2^{*}$ and $q_2^{*}=p_1^{*}$. Thus, an erroneous action means that the probabilities are restored: in that anomalous game the perturbed BR player actually uses the ESS. The case of BR opponent can be treated analogously. In the latter case $W(\beta ^{\prime }, \beta ^{\prime })=W(\beta ,\beta )$, because the strategies of the two best reply opponents remain independent and uniform. By the properties of the mixed ESS, $W(\beta ^{\prime },p^{*})=W(p^{*},p^{*})=W(\beta ,p^{*})$. Finally, in the repeated games $W(p^{*},\beta ^{\prime })=(1-\delta ) W(p^{*},\beta )+\delta W(p^{*},p^{*})$. This is a little bigger than $W(p^{*},\beta )$, but still less than $W(\beta ,\beta )=W(\beta ^{\prime }, \beta ^{\prime })$ if $\delta$ is sufficiently small. Thus, the dominance of the best reply strategy can be preserved.

Observe that in the present case, the best reply player has two-step behavior cycles; thus, if each pair repeats the interaction with its opponent only once again, then the advantage of best reply players can already occur. Thus, if the one-shot ESS is a mixed strategy, it is not necessarily evolutionarily stable in the two-round game. This advantage may even remain in the case where the average number of (random) repetitions is arbitrarily small as shown in the next section.

We emphasize that the BR behavior is one of the simplest action-reaction rules, since the BR player can only remember the opponent’s pure strategy used in the previous game, but cannot remember who won, for example. Therefore, phenomena such as the loser-winner effect leading to some kind of “asymmetry” (like dominance) cannot emerge in our iterated game (Morrell and Kokko 2003). Consequently, our game is symmetric, since all players either use the mixed ESS or the BR strategy, and there are no more differences among them.

### Well-Mixed Games with Repetition: A Particular Case

**Fig. 1:**
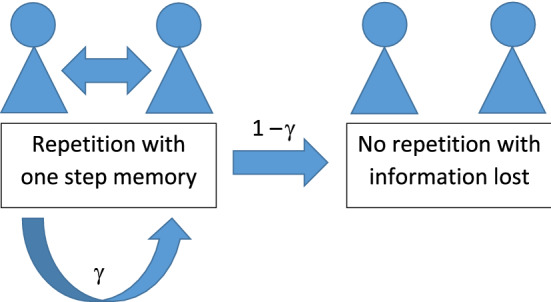
Well-mixed game with repetition. In the figure $\gamma$ denotes the probability that the given pair repeats the game being a matrix game with payoff matrix $A=(a_{ij})_{2\times 2}$, where $a_{11}=c$, $a_{12}=b$, $a_{21}=c+a$, $a_{22}=0$. If $\gamma =0$, then no repetition takes place, that is, pair formation is governed by random mixing. If $\gamma =1$, then the game is iterated, that is, the pairs repeat the game arbitrarily many times. In this case, if $c>\frac{3}{2}(b-a)$, the best reply (BR) player phenotype replaces the mixed evolutionarily stable strategist (ESS) phenotype. If the game is repetitive, that is, playing pairs can, but not necessarily have to, repeat the game, and $c>\big (1+\frac{1}{2\gamma }\big )(b-a)$, then for every $\gamma \in (0,1)$ the parameters *a*, *b*, *c* of the payoff matrix can be chosen in such a way that BR players can replace those following a mixed ESS. In order that the cases of iterated and repeated games can easily be compared we suppose that the players cannot distinguish their opponents, they only know if the next game will be played with the same opponent, and, in that case, what strategy was used by the old-new opponent in the preceding round. Thus, if a BR–BR pair splits up, but they get together again during the well-mixed pair formation process, then both BR players consider the other one a newcomer. This simplifying condition obviously favors the ESS players, as it decreases the number of cycles in BR–BR pairs

As we have already mentioned in Sect. 1, iterated games have properties very different from those of one-shot games. Now we are interested in the selection situation where well-mixing interactions and game repetitions occur in a population at the same time (see Fig. 1).

Suppose we have a population of size *N*, where *N* is a sufficiently large even number. In the population, pairwise interactions take place; in these interactions a matrix game is played by the pair (the same game at every instance). For the sake of simplicity, we focus on the $2\times 2$ example we dealt with in Sect. 2.1. Time is considered discrete; that is, the history of the population evolves in separate turns. There are two phenotypes present in the population. They are characterized by the strategy they use in the matrix game: phenotype $p^{*}$ follows a mixed ESS strategy (which is assumed to exist), and phenotype $\beta$ is the best reply player. There are $(1-\varepsilon )N$ and $\varepsilon N$ of them, respectively (these numbers are even integers). In every turn, there may be pairs who randomly stick together for a repeated game. All the other pairs split up, and the free individuals are to be organized in new pairs for the next round. Pair formation is supposed to be completely random and independent of the past. The event that a given pair keeps together is independent of the past and also of other pairs. The probability of repetition is denoted by $\gamma \in (0,1)$.

When a pair plays their first game, a best reply player choses her strategy uniformly at random, but in the subsequent repetitions she applies the pure strategy being a best reply against the action of the opponent in the previous round. Even if a pair splits, they can happen to reunite in the next turn. Should it be the case, the best reply player is supposed to forget the last turn and randomize their strategy as if a new opponent were met. Note that this possibility is very unlikely for large *N*; thus, the effect of this choice is negligible.

#### Theorem 1

Let *I* denote the open interval with endpoints *a* and $a+c$, and let ${{{\bar{I}}}}$ be its closure. Moreover, define

$$\begin{aligned} Q=\frac{b-a}{2[c-(b-a)]}. \end{aligned}$$

- If $b\in I$, and $\gamma >Q$, then the best reply strategy displaces the ESS if *N* is large enough.
- On the other hand, if $b\notin {{{\bar{I}}}}$ or $b\in I$ but $\gamma <Q$, then the ESS displaces the best reply strategy.

Note that $b\in I$ implies $Q>0$. With $\gamma =1$, we get back the result of Sect. 2.1. In the numerical example of Sect. 2.1 as small as $\gamma =1/3$ already ensures the best reply player’s domination. This only means half a repetition on average.

#### Proof

We will prove that in the limit as $N\rightarrow \infty$ the average payoff of a BR player is greater or less than that of an ESS player, independently of the relative frequency $\varepsilon$ of BR players, according that the conditions in (a) or (b) are satisfied, resp.

Let $Z_n$ denote the number of $(\beta ,\beta )$ pairs in round *n*. Then, $(Z_n, \,n=1,2,\dots )$ is a homogeneous Markov chain with state space

$$\begin{aligned} \textstyle {{\mathcal {S}}}=\left\{ k\ge 0: \left( \varepsilon -\frac{1}{2}\right) N \le k \le \frac{1}{2}\varepsilon N\right\} . \end{aligned}$$

The state space can be obtained in the following way. Clearly, $2k\le \varepsilon N$ on the one hand. On the other hand, in addition to these *k* pairs, there are $\varepsilon N-2k$ of BR players and $(1-\varepsilon )N$ of ESS players to be coupled. Then $(1-\varepsilon )N\ge \varepsilon N-2k$, or equivalently, $\left( \varepsilon -\frac{1}{2}\right) N\le k$ is needed, otherwise further $(\beta ,\beta )$ pairs must be formed.

It is thus clear that if there are *k* of $(\beta ,\beta )$ pairs, then the number of $(p^{*},\beta )$ pairs is $\varepsilon N-2k$ and we also have $\left( \frac{1}{2} -\varepsilon \right) N+ k$ pairs of type $(p^{*},p^{*})$. Since the number of $(\beta ,\beta )$ pairs determines the composition of all pairs, and this composition determines the joint distribution of the number of different pair types in the next round, the Markov property of the sequence $Z_n$ follows.

Formally, the transition probabilities can be found in Appendix. (These formulas will not be needed in the sequel.)

This Markov chain is irreducible and aperiodic, because all states communicate with each other in a single step: it can happen with positive probability that all pairs split up and reunite in an arbitrarily prescribed way. Thus, there exists a unique equilibrium distribution on ${{\mathcal {S}}}$; this is the asymptotic distribution of the chain as the number of turns tends to infinity, irrespectively of the initial distribution. For a Markov chain like that the strong law of large numbers is valid, the time average of an arbitrary function $f:{{\mathcal {S}}}\rightarrow {{\mathbb {R}}}$ of the states tends almost surely to the expectation of *f* in the stationary regime.

Whenever two players meet, they play a random number of games, which is geometrically distributed with mean $\frac{1}{1-\gamma }$. Let us denote the average per game payoff of each participant of a $(\beta , \beta )$ type game sequence by $W(\beta ,\beta )$. Considering a pair of type $(p^{*},\beta )$, the average per game payoff for phenotypes $p^{*}$ and $\beta$ will be denoted by $W(p^{*},\beta )$ and $W(\beta ,p^{*})$, resp. Finally, let $W(p^{*},p^{*})$ denote the average per game payoff of each player of a $(\beta , \beta )$ pair during this sequence of repeated games. Then $W(\beta ,p^{*})=W(p^{*},p^{*})$ because the ESS $p^{*}$ is totally mixed, i.e., it assigns positive probabilities to all pure strategies, as in this case $q\,A\,p^{*}=p^{*}A\,p^{*}$ must hold for all $q\in \Sigma _{d}$.

Suppose we are in the stationary regime. Let *z* be equal to the expectation of the stationary distribution, divided by *N*. Let the number of $(\beta ,\beta )$ pairs be equal to *Z*, then there are $\varepsilon N-2Z$ pairs of type $(p^{*},\beta )$ and $\left( \frac{1}{2}-\varepsilon \right) N+Z$ pairs of type $(p^{*},p^{*})$. Introduce $z=2E(Z)/N$. The average payoff for an ESS player is

4 $$\begin{aligned} {{\overline{W}}}(p^{*})=:\frac{1}{1-\varepsilon }\left[ (\varepsilon -z)W(p^{*},\beta ) +(1-2\varepsilon +z)W(p^{*},p^{*})\right] . \end{aligned}$$

Furthermore, the average payoff for a best reply player is

5 $$\begin{aligned} {{\overline{W}}}(\beta )=:\frac{1}{\varepsilon }\left[ (\varepsilon -z)W(\beta ,p^{*})+ z\,W(\beta , \beta )\right] . \end{aligned}$$

Let us compute *z*, at least asymptotically as $N\rightarrow \infty$. We remark here that Benaïm and Weibull (2003) deal with the goodness of deterministic approximations of Markovian stochastic population processes that arise from individual strategy adaptation in finite but large populations. Their general results support the validity of our large *N* approximations. Note, however, that in their paper the term best reply strategy is used in another sense: not in the context of pairwise interactions, but rather as best reply to the current population state which is supposed to be observable by the player.

If in a round there are *Z* pairs of type $(\beta ,\beta )$, an average number $Nz\gamma /2$ of them will stay together. On the other hand, $N(\varepsilon -z)(1-\gamma )$ pairs of type $(p^{*},\beta )$ will split up on the average. Thus, there will be $N(1-\gamma )$ free individuals seeking a pair in the next round, with $N\varepsilon (1-\gamma )$ of them belonging to phenotype $\beta$. Now, the mean number of $(\beta ,\beta )$ pairs they form is asymptotically equal to $N\varepsilon ^{2}(1-\gamma )/2$ for large *N*. Being in equilibrium, one obtains the following equation for *z*:

$$\begin{aligned} z=z\gamma +\varepsilon ^{2}(1-\gamma ), \end{aligned}$$

whence $z=\varepsilon ^{2}$. Plugging it back into (4) and (5), we get that the average payoff for an ESS player is

$$\begin{aligned} {{\overline{W}}}(p^{*})= & {} \frac{1}{1-\varepsilon }\big [(\varepsilon -\varepsilon ^2) W(p^{*},\beta )+(1-2\varepsilon +\varepsilon ^2)W(p^{*},p^{*})\big ]\\= & {} \varepsilon \,W(p^{*},\beta )+(1-\varepsilon )W(p^{*},p^{*}), \end{aligned}$$

while the same for a best reply player is

$$\begin{aligned} {{\overline{W}}}(\beta )=\frac{1}{\varepsilon }\big [(\varepsilon -\varepsilon ^2) W(\beta ,p^{*})+\varepsilon ^2 W(\beta ,\beta )\big ] =(1-\varepsilon )W(\beta ,p^{*})+\varepsilon \,W(\beta ,\beta ). \end{aligned}$$

Since $W(\beta ,p^{*})=W(p^{*},p^{*})$, we obtain that

6 $$\begin{aligned} {{\overline{W}}}(p^{*})<{{\overline{W}}}(\beta )\quad \text{ if } \text{ and } \text{ only } \text{ if }\quad W(p^{*},\beta )<W(\beta ,\beta ). \end{aligned}$$

In Sect. 2.1, we have already seen that

$$\begin{aligned} W(\beta , \beta )=\frac{a+b+2c}{4}\,. \end{aligned}$$

In this case, the average payoff does not vary during the sequence of repeated games. The case of $W(p^{*},\beta )$ is somewhat different. The payoff for phenotype $p^{*}$ in a pair of type $(p^{*},\beta )$ is

$$\begin{aligned} w'(p^{*},\beta )=:\frac{ac+bc+a^{2}+b^{2}}{2(a+b)} \end{aligned}$$

in the first game, and

$$\begin{aligned} w''(p^{*},\beta )=:\frac{ca}{a+b}+\frac{a^{3}+b^{3}}{(a+b)^{2}}= \frac{ac-ab+a^{2}+b^{2}}{a+b} \end{aligned}$$

in further games. Altogether this is

$$\begin{aligned} W(p^{*},\beta )&=(1-\gamma )\left[ w'(p^{*},\beta )+w''(p^{*},\beta ) \cdot \frac{\gamma }{1-\gamma }\right] \\&=(1-\gamma )\cdot \frac{ac+bc+a^{2}+b^{2}}{2(a+b)}+\gamma \cdot \frac{ac-ab+a^{2}+b^{2}}{a+b}\\&=\frac{ac+bc+a^{2}+b^{2}}{2(a+b)}+\gamma \cdot \frac{(b-a)(c+a-b)}{a+b} \end{aligned}$$

on the average.

Thus, the BR strategy is more fruitful than the ESS if

$$\begin{aligned} \frac{ac+bc+a^{2}+b^{2}}{2(a+b)}+\gamma \cdot \frac{(b-a)(c-b+a)}{a+b} <\frac{a+b+2c}{4}\,. \end{aligned}$$

Multiplying this inequality by $4(a+b)$ and rearranging, we get the condition

$$\begin{aligned} 2\gamma (b-a)(c-b+a)>(b-a)^2. \end{aligned}$$

If $(b-a)(c-b+a)<0$, that is, $b\notin {{{\bar{I}}}}$, then the left-hand side is negative while the right-hand side is positive, thus the opposite inequality holds: the ESS outperforms the BR strategy. If $(b-a)(c-b+a)>0$, that is, $b\in I$, then we can divide both sides by it, and the condition for the dominance of the BR strategy reads $\gamma >Q$. Again, the opposite inequality implies the dominance of the ESS.

This was obtained by replacing *z* with $\varepsilon ^2$, which, in fact, is the limit of *z* as $N\rightarrow \infty$. If inequality $\gamma >Q$ (or the opposite inequality) holds, then the limit of ${{\overline{W}}}(p^{*})$ is strictly less than (or greater than) that of ${{\overline{W}}}(\beta )$, therefore the same inequality must hold whenever *N* is sufficiently large. $\square$

**Fig. 2:**
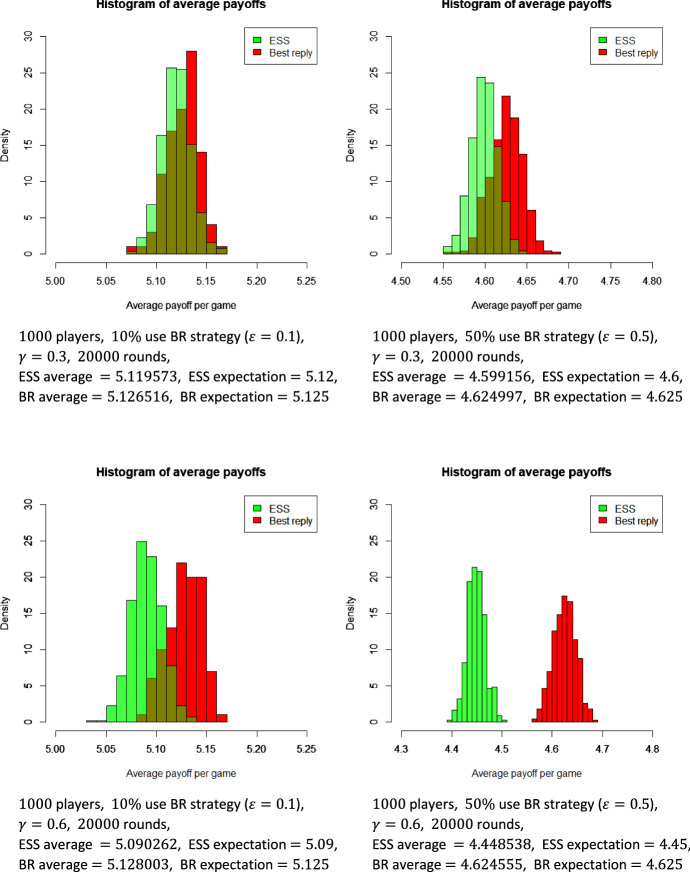
Simulations. In the diagrams two values of the repetition probability $\gamma$ are combined with two values of the density $\varepsilon$ of the BR players. In all four cases the population size is equal to 1000, and the number of rounds is 20000. The payoff matrix is also fixed: $a_{11}=6$, $a_{12}=3$, $a_{21}=4$, $a_{22}=0$. The green (light gray) histogram illustrates the distribution of per game average payoffs for the ESS players. The red (dark grey) one is the same for BR players. The two histograms diverge more and more as the probability of repetition or the proportion of BR players grows, implying that the advantage of the best reply strategy is getting more and more significant (Color figure online)

We have performed simulations to illustrate the speed of convergence of empirical data to the theoretical results in the numerical example of Sect. 2.1. The program codes were written in *R*. In Fig. 2, four diagrams illustrate the effect of the parameters $\gamma$ and $\varepsilon$ on the difference between ESS and BR players the per game average payoff. Two values of the repetition probability ($\gamma =0.3$ and $\gamma =0.6$) are combined with two values of the BR density ($\varepsilon =0.1$ and $\varepsilon =0.5$). In all four cases the population size is equal to 1000, and the number of rounds (games) is 20000. The payoff matrix is $$A=\left( {\begin{matrix} 6 &{} 3 \\ 4 &{} 0 \end{matrix}}\right) $$. The green histogram illustrates the distribution of per game average payoffs for the ESS players. The red one is the same for BR players. The two histograms are getting further and further apart as the probability of repetition or the proportion of BR players grows, implying that the advantage of the best reply strategy is getting more and more significant. Note that a repetition probability as small as $\gamma =0.3$ already ensures the best reply player’s dominance. This means less than half repetitions on average. When $\gamma =0.6$, the expected number of repetitions is $\frac{\gamma }{1-\gamma }=1.5$, still rather small.

### Well-Mixed Games with Repetition: The General Case

Theorem 1 can easily be generalized to more complex games with larger matrices. Suppose the matrix game is defined by an $d\times d$ nonnegative invertible matrix *A* and there exists a totally mixed ESS $p^{*}$, i.e., every coordinate of $p^{*}$ is positive.

For $j=1,\dots ,d$ let $\sigma (j)$ be the row of the maximal element in column *j* (one of them, if there are more), i.e., $\max _{1\le i\le d}a_{i,j} =a_{\sigma (j),j}$. In other words, pure strategy $s_{\sigma (j)}$ is a/the best reply to pure strategy $s_j$. Let $\sigma ^0(j)=j$ for all $j=1,\dots ,d$, and for $k=1,2,\dots$ define $\sigma ^{k}= \sigma \circ \sigma ^{k-1}$.

When a BR player is paired with a new partner, in the first game she selects from the pure strategies uniformly at random, then in every repeated game chooses her strategy according to the function $\sigma$.

#### Theorem 2

Let $$e=(1,\dots ,1)\in {{\mathbb {R}}}^n$$. Suppose

7 $$\begin{aligned} (1-\gamma )\cdot \frac{1}{d}\,p^{*}A\,e+\gamma \,\sum _{i=1}^d\,\sum _{j=1}^d p_i^{*}p_j^{*} a_{i,\sigma (j)}< \frac{1}{d^2}\,\sum _{k=0}^{\infty }{\gamma }^k(1-\gamma )\sum _{i=1}^d\, \sum _{j=1}^d a_{\sigma ^{k}(i),\sigma ^{k}(j)} \end{aligned}$$

holds. Then, the best reply strategy displaces the ESS if *N* is large enough. On the other hand, if in (7) the opposite (strict) inequality is valid, then the ESS displaces the best reply strategy for every sufficiently large *N*.

#### Proof

The proof of Theorem 1 up to the formula (6) does not utilize the particular form of the payoff matrix *A*, hence (6) remains true in the present case. What we only need is to compute $W(p^{*},\beta )$ and $W(\beta ,\beta )$.

When an ESS player is paired with a BR player, in the first game the latter chooses her action uniformly at random; therefore, the average payoff of the ESS player is $\frac{1}{d} p^{*}A\,e$. In every subsequent round, if the ESS player chooses strategy $s_i$ and in the preceding round she chose $s_j$, then her opponent plays $s_{\sigma (j)}$ and thus her payoff is $a_{i,\sigma (j)}$. This occurs with probability $p_i^{*}p_j^{*}$. Hence, the average payoff per game is

$$\begin{aligned} \sum _{i=1}^d\,\sum _{j=1}^d p_i^{*}p_j^{*} a_{i,\sigma (j)}, \end{aligned}$$

and the expected number of such games is $\gamma /(1-\gamma )$. Thus, the left-hand side of (7) is just the per game average payoff of the ESS player.

When two BR players form a pair, in the first game they choose their strategies independently and uniformly at random. Suppose they choose $s_i$ and $s_j$. Then, in the $k+1$-st game they play $s_{\sigma ^k(i)}$ and $s_{\sigma ^k(j)}$, resp., if *k* is even, and $s_{\sigma ^k(j)}$, $s_{\sigma ^k(i)}$ if *k* is odd. Thus, each receives an average payoff of

$$\begin{aligned} \frac{1}{d^2}\,\sum _{i=1}^d\,\sum _{j=1}^d a_{\sigma ^{k}(i),\sigma ^{k}(j)}, \end{aligned}$$

provided they are still together in the $k+1$-st game. This happens with probability $\gamma ^k$. The multiplier $1-\gamma$ appearing in the right-hand side of (7) performs averaging per game. $\square$

Condition (7) is not so convenient to check in general. However, in the following special case it becomes very simple. Remember that the ESS $p^{*}$ is supposed to be totally mixed. In this case thus the row sums of $A^{-1}$ are all positive and $p^{*}= (e\,A^{-1}e)^{-1}A^{-1}e$.

#### Corollary 3

Suppose that

(i) $\sigma$ is a permutation of $\{1,2,\dots ,d\}$.
(ii) $$e\,Ae>d^{2}(p^{*}A\,q^{*})$$, where $$q^{*}_{\sigma (i)}=p^{*}_{i}$$, $$1\le i \le d$$.
(iii) $$\displaystyle \gamma >\frac{d^{-1}p^{*}A\,e-d^{-2}eA\,e}{d^{-1}p^{*}A\,e-p^{*} A\,q^{*}}=\frac{d\,p^{*}A\,e-eA\,e}{d\,p^{*}A\,e-d^{2}p^{*}A\, q^{*}}\,.$$

Then, the BR strategy eventually replaces the ESS if *N* is large enough.

#### Proof

Since $p^{*}$ is a totally mixed ESS, $d^{-1}p^{*}A\,e>d^{-2}e\,A\,e$ must hold. Combining this with condition (ii) we can see that the fraction in the right-hand side of (iii) has positive numerator and denominator.

By the definition of $q^{*}$,

$$\begin{aligned} \sum _{i=1}^d\,\sum _{j=1}^d p_i^{*}p_j^{*} a_{i,\sigma (j)}= \sum _{i=1}^d\,\sum _{j=1}^d p_i^{*}q_j^{*} a_{i,j}=p^{*}A\,q^{*}. \end{aligned}$$

Moreover, $\sigma ^k$ is a permutation for every $k=0,1,\dots$, hence

$$\begin{aligned} \sum _{i=1}^d\,\sum _{j=1}^d a_{\sigma ^{k}(i),\sigma ^{k}(j)}= \sum _{i=1}^d\,\sum _{j=1}^d a_{i,j}=e\,A\,e. \end{aligned}$$

Thus condition (iii) is equivalent to (7). $\square$

Note that the right-hand side of (iii) is less than 1 by condition (ii), hence conditions (i)–(ii) always make it possible for the best reply player to outperform the ESS in a finite mean random number of repetitions.

### Modification of the Best Reply Strategy

Finally, the following question also arises: If in the game considered above the interaction is well mixed, can the best reply player invade a monomorphic classical mixed ESS population? Clearly, when each best reply player has a randomly chosen new opponent from round to round, then the deterministic behavior cycle of a pair of best reply players does not occur, and the ESS strategy remains better than the uniform random strategy used by the best reply players.

Let us modify the best reply behavior in such a way that the best reply player’s next strategy always depends on the strategy her random opponent used in the previous round, even if her opponent has changed. Let us denote this modified strategy (and the corresponding phenotype) by $\beta ''$. This time we suppose that the pair formation is well mixed: all pairs split up and the players reunite in a completely random way. In the very beginning the modified BR players choose their strategies uniformly at random. Like we did in Sect. 2.3, let us fix $\sigma (i)$ again for every pure strategy $s_i\in S$ in such a way that $s_{\sigma (i)}$ is a best reply to $s_i$. The modified BR player uses strategy $s_{\sigma (i)}$ if $s_i$ was the strategy her partner used in the previous game. The modified BR strategy $\beta ''$ is better than the ESS if the per game and player average of the payoff of $\beta ''$ strategists exceeds that of the ESS players in the long run. In that case the modified BR strategy can replace the ESS.

#### Theorem 4

In a sufficiently large population, the modified best reply strategy cannot displace the (totally) mixed ESS.

#### Proof

In course of the consecutive well-mixed rounds, each best reply player’s strategy series is a random sequence of not necessarily independent pure strategies. Since each well-mixed round determines the next strategy distribution of the best reply players, we can introduce a discrete time Markov chain to describe the strategy distribution of the subpopulation of best reply players.

For a formal definition let *H* denote the set of indices of pure strategies $s_{i}$ that are best replies to certain pure strategies, and for $i\in H$ let ${\tau }_{i}$ be any pure strategy against which $s_{i}$ is optimal. (They do not need to be all different.) Let *N* denote the size of the population; we suppose that *N* is a sufficiently large even number. The states of the discrete time stochastic process $\kappa _2,\kappa _3,\dots$ are $\left| H\right|$ dimensional vectors $(k_{i})_{i\in H }$ with nonnegative integer coordinates summing up to $\varepsilon N$, the number of modified best reply players. Formally, the state space is

$$\begin{aligned} {{\mathcal {K}}}=\left\{ {{\mathbf {k}}}\in {{{\mathbb {N}}}}^H: {\textstyle \sum _{i\in H} k_{i}= \varepsilon N}\right\} , \end{aligned}$$

and $\kappa _n$ is the random vector taking its values from ${{\mathcal {K}}}$ whose *i*-th coordinate is the number of best reply players playing strategy $s_i$, $i\in H$ in the *n*-th round. ($n>1$ is needed because in the first round best reply players still not necessarily use best reply strategies.) We will show that this process is a homogeneous Markov chain.

Let $\mu _2,\mu _3,\dots$ be another stochastic process with the *d* dimensional state space

$$\begin{aligned} {{\mathcal {M}}}=\left\{ {{\mathbf {m}}}\in {{{\mathbb {N}}}}^d: {\textstyle \sum _{i=1}^d m_{i}= (1-\varepsilon ) N}\right\} , \end{aligned}$$

defined as follows. Let the *i*-th coordinate of $\mu _n$ be the number of ESS players playing strategy $s_i$, $1\le i\le d$ in the *n*-th round. Then, $\mu _1,\mu _2,\dots$ are identically distributed according to the *d*-variate multinomial distribution with parameters $(1-\varepsilon )N$ and $p^{*}$. Moreover, $\mu _n$ is independent of $\kappa _2,\mu _2,\dots ,\kappa _{n-1},\mu _{n-1},\kappa _n$. The pair $(\kappa _n,\mu _n)$ determines the strategy frequencies in the *n*-th round. It is clear that the process $(\kappa _n,\mu _n)$, $n=2,3,\dots$, is a Markov chain.

We will use the following elementary fact. Let $C_1,C_2,\dots$ be a partition of random events, which is independent of the event *B*. Then, by the law of total probability

$$\begin{aligned} P(A\mid B)=\sum _i P(A\mid B\cap C_i)P(C_i\mid B)=\sum _i P(A\mid B\cap C_i)P(C_i). \end{aligned}$$

By using this formula twice, we get

$$\begin{aligned} P(\kappa _n&={{\mathbf {k}}}_n\mid \kappa _{n-1}={{\mathbf {k}}}_{n-1}, \dots ,\kappa _2= {{\mathbf {k}}}_2)\\&=\sum _{{{\mathbf {m}}}\in {{\mathcal {M}}}} P(\kappa _n={{\mathbf {k}}}_n\mid \kappa _{n-1}= {{\mathbf {k}}}_{n-1},\dots ,\kappa _2={{\mathbf {k}}}_2,\mu _{n-1}={{\mathbf {m}}}) P(\mu _{n-1}={{\mathbf {m}}})\\&=\sum _{{{\mathbf {m}}}\in {{\mathcal {M}}}} P(\kappa _n={{\mathbf {k}}}_n\mid \kappa _{n-1}= {{\mathbf {k}}}_{n-1},\mu _{n-1}={{\mathbf {m}}})P(\mu _{n-1}={{\mathbf {m}}})\\&=P(\kappa _n={{\mathbf {k}}}_n\mid \kappa _{n-1}={{\mathbf {k}}}_{n-1}), \end{aligned}$$

as needed.

Now the question is whether this Markov process is irreducible or not. In our case when ESS players use well-mixed strategies (that put positive weight on each possible pure strategy), and the interaction is well mixed (that is, each pairing of individuals for interaction is equally possible), this Markov process is irreducible and aperiodic, provided $\varepsilon \le 1/2$. Indeed, the transition probability between every pair of states is positive, because it can happen with positive probability that each best reply player interacts with an ESS player, and these opponents follow strategy ${\tau }_{i}$ $(i\in H)$ just in as many occasions as needed for getting to the object state.^3^ Thus there exists a unique stationary distribution; let us denote it by $\{\pi _{{{\mathbf {k}}}},{{\mathbf {k}}}\in {{\mathcal {K}}}\}$. This is also the asymptotic distribution of the Markov chain as the number of turns tends to infinity. $\{\pi _{{{\mathbf {k}}}}\}$ is a probability measure on the state space; $\left| H\right|$-variate, for the state space itself is $\left| H\right|$-dimensional. If the number of turns tends to infinity, the average number of best reply players following strategy $i\in H$ is asymptotically $\sum _{{{\mathbf {k}}}\in {{\mathcal {K}}}} k_i\pi _{{{\mathbf {k}}}}$. In the long run, the vector of average proportions of pure strategies followed by best reply players is just the expectation of the stationary distribution, divided by the number $\varepsilon N$ of such players. Denote this vector by $q^{*}\in {\Sigma }_{\left| H\right| }$, thus $q^{*}_{i}$ is the average proportion of best reply players playing strategy $s_{i}$. That is,

$$\begin{aligned} q_{i}^{*}=\frac{\sum _{{{\mathbf {k}}}\in {{\mathcal {K}}}} k_i\pi _{{{\mathbf {k}}}}}{\sum _{{{\mathbf {k}}}\in {{\mathcal {K}}}} k_i}=\frac{1}{\varepsilon N} \sum _{{{\mathbf {k}}} \in {{\mathcal {K}}}} k_i\pi _{{{\mathbf {k}}}}. \end{aligned}$$

This is a probability distribution on *H*, because

$$\begin{aligned} \sum _{i\in H}q_{i}^{*}=\frac{1}{\varepsilon N}\sum _{i\in H} \sum _{{{\mathbf {k}}} \in {{\mathcal {K}}}} k_i\pi _{{{\mathbf {k}}}} =\frac{1}{\varepsilon N}\sum _{{{\mathbf {k}}} \in {{\mathcal {K}}}}\pi _{{{\mathbf {k}}}} \sum _{i\in H} k_i=\sum _{{{\mathbf {k}}}\in {{\mathcal {K}}}}\pi _{{{\mathbf {k}}}}=1. \end{aligned}$$

Let us extend $q^{*}$ to the set of all pure strategies by setting $q_{i}^{*}=0$ for $i\notin H$.

The existence of this stationary strategy distribution gives an easy way to calculate the average payoff of phenotypes; thus, we have $W(p^{*},p^{*})= p^{*}A\,p^{*}$, $W(p^{*},\beta '')=p^{*}A\,q^{*}$, $W(\beta '',p^{*})=q^{*}A\,p^{*}$ and $W(\beta '',\beta '')= q^{*}A\,q^{*}$. Hence (2) can be applied, consequently the mixed ESS $p^{*}$ remains stable. Note that in the long run, the distribution of pure strategies followed by each individual $\beta ''$ player is also equal to $q^{*}$. $\square$

## Discussion and Conclusions

In summary, strictly following the intuitive definition of evolutionary stability (1) by Maynard Smith and Price (1973), we found the following results.

Firstly, we gave an example where the classical mixed ESS $p^{*}$ defined by (2) is not evolutionary stable in the framework of iterated $2\times 2$ matrix games. The novelty of the present paper is that here the mixed ESS can be replaced by a BR player. The mixed ESS is a Nash equilibrium which, when playing with a best reply opponent, receives a larger payoff in every round than another best reply player. Nonetheless, we have shown that best reply players can achieve a larger average payoff than players following the ESS. This is possible because best reply players in pairs, individually following their own strategies, develop cycles where the bigger payoff can compensate their disadvantage compared with the ESS players. This phenomenon is independent of the proportions of types in the population; that is, not affected by the frequencies of different pair types. It can even occur when the mean number of repetitions is arbitrarily small and it can also tolerate a small chance of mistake.

Secondly, based on the above observation, we also showed that in the well-mixed population game with repetition the classical mixed ESS loses its overall evolutionary stability. In more details, if there is but a small probability that two players repeat the matrix game, then, depending on the payoff matrix, it can occur that the BR player outperforms the classical mixed ESS.

Mixed or reactive strategy? Maynard Smith wrote in his book (1982): *“Animals do not have roulette wheels in their hands”*, and he offered genetic and developmental mechanisms, which can give rise to variable behavior.^4^ We note that individual-based simulations called the attention to the fact that if the behavioral strategies are either implemented by a 1 : 1 genotype–phenotype mapping or by a simple neural network, the evolutionary outcomes are different, largely depending on the behavioral and genetic architecture (van den Berg and Weissing 2015). Moreover, it seems that humans can generate random numbers that are uniformly distributed, independent of one another and unpredictable (Persaud 2005). However, a number of studies have shown that people often deviate slightly from the prediction of the classical game theory (see Barraclough et al. 2004; Wright and Leyton-Brown 2010, and the references therein). On the other hand, as we have mentioned in Sect. 1, when the individuals are supposed to live in a small group, the iterated prisoner’s dilemma game is often used in evolutionary biology, where the evolutionary success of reactive strategies (like “tit for tat” ) over the pure defector is investigated. Moreover, since real populations are finite, the possibility of game repetition cannot be neglected, thus considering reactive players seems reasonable against mixed ESS. The basic intuition behind both research lines (pure or mixed ESS) rests upon the fact that in evolutionary game theory there is no biological reason to consider only nonreactive mixed strategy users as phenotypes. Finally, the overwhelming majority of biologists agree that living things can respond to stimuli, thus considering reactive players is biologically reasonable.

Although our paper strictly belongs to evolutionary game theory, we would like to call the attention to possible connections with classical game theory. The evolutionary success of BR is based on the assumption that the games are iterated and well mixed with repetition. In the iterated noncooperative games, the following question is quite important (Camerer and Ho 1999): “Which models describe human behavior best?” One possibility is the best-response (Camerer and Ho 1999), and the other one is the learning (Camerer et al. 2003), and both models are based on the belief about what others will do in the future based on past observation. Furthermore, the BR is not only a possible human behavior, but it also give a method to answer another basic question (Camerer and Ho 1999): “How does an equilibrium arise in a noncooperative game?” The BR also help to reach the equilibrium (Ho et al. 1998). Although we only pointed out that the mixed ESS can easily lose its stability against the reactive player, we hope our result may be of interest to game theorists in the field of mathematical economics.

Based on Sects. 2.1 and 2.2, it seems that evolutionary stability of a mixed strategy occurs when the interactions are well mixed in a large enough population (see also Bendor and Swistak 1995; Boyd and Lorberbaum 1987; Farrell and Ware 1989; Garay and Varga 2011; García and van Veelen 2016). There are essentially two ways of weakening this condition. In the first way, the interaction between the same phenotype is more likely. Observe that here the repetition of a game is not a must. For instance, if the probability of interaction between the same phenotype is small (e.g., game between relatives Hines and Maynard Smith 1979), then the classical ESS changes but a little. In other words, for a small change the mixed ESS is structurally stable. However, we note that if the clonal interaction rate is high enough, then that phenotype will win in the natural selection which maximizes the average fitness of its clone (Garay and Varga 2011).

In the second way, the players can repeat the game with a certain probability. Our main result is that in this way the classical mixed ESS loses its overall evolutionary stability, since even a small change can make the BR player able to outperform it. This is only true if the payoff matrix is not fixed: in a fixed game the winning probabilities of repetition are bounded away from zero, but for an arbitrarily small positive probability of repetition one can find payoff matrices so that the classical mixed ESS loses its advantage. This may be called weak structural instability.

## Appendix A

In this Appendix, we provide the transition probabilities of the Markov chain $(Z_n)$. Let $$\varrho _i(m)=\left( {\begin{array}{c}m\\ i\end{array}}\right) (1-\gamma )^i\gamma ^{m-i}$$; the *i*-th term of the binomial distribution with parameters *m* and $1-\gamma$. This is the probability that exactly *i* out of *m* pairs would split up. Let $\kappa (m)$ denote the number of all possible arrangements of 2*m* players into *m* pairs, i.e., $\kappa (m)=\frac{(2m)!}{2^m m!}$. Finally, let $\lambda _m(u,v)$ be the probability that, randomly arranging *u* red and *v* white balls ($u+v$ is even) in pairs, exactly *m* pairs of (red, red) type are formed $(m\le \frac{u}{2})$. Then

$$\begin{aligned} \lambda _m(u,v)=\left( {\begin{array}{c}u\\ u-2m\end{array}}\right) \left( {\begin{array}{c}v\\ u-2m\end{array}}\right) (u-2m)!\frac{\kappa (m)\kappa ( \frac{v-u}{2}+m)}{\kappa (\frac{u+v}{2})}\,. \end{aligned}$$

Let $\lambda _m(u,v)=0$ if $m<0$ or $m>\frac{u}{2}$.

Suppose we have $N_1=k$ pairs of $(\beta ,\beta )$ type, $N_2=\varepsilon N-2k$ pairs of $(p^{*},\beta )$ type and $N_3=\left( \frac{1}{2} -\varepsilon \right) N+ k$ pairs of $(p^{*},p^{*})$ type. They split up and rearrange at random. Let *i*, *j*, and $\ell$ denote the number of splitting pairs, respectively. Then,

$$\begin{aligned} P\left( Z_n=m\mid Z_{n-1}=k\right) =\sum _{i=0}^{N_1}\,\sum _{j=0}^{N_2}\, \sum _{\ell =0}^{N_3}\varrho _i(N_1)\varrho _j(N_2)\varrho _{\ell }(N_3) \lambda _{m-k+i}(2i+j,2\ell +j). \end{aligned}$$

## Footnotes

1 For the “dynamical player” a game-dynamics solution concept is the following: a strategy pair (or a behavior cycle) is a dynamic solution if it is a locally asymptotically stable rest point with respect to a game dynamics describing the behavior of the players (Garay et al. 2015).

2 The simplest one step memory excludes the possibility of considering more sophisticated reaction rules (see, e.g., Ohtsuki and Iwasa 2006; Wolf et al. 2011).

3 If the ESS is not well mixed though the interaction is well mixed, the above proof of irreducibility breaks down. What can be said in that case is an open problem.

4 There are some papers where a controversy arose if a mixed strategy can be an ESS. Maynard Smith (1988) and Vickery (1987) were involved in the discussion. See also (Schaffer 1988).
